# 

**Published:** 1890-07

**Authors:** 


					﻿JULY, 1890,
OLD SERIES
Vol. XXV
NEW SERieS
Vol. VII.—No. 5.
& 1855 O

HEDIGMs^ oURi^^s
JDURNAK



w. F. WESTMORELAND, M.D.
H. V. M. MILLER, M.D., LL.D.
VIRGIL 0. HARDON, M.D.
F. W. McRAE, M.D., M’n’g’ng Ed.
^IIRLISHED By james p.harrison&.co:j
/CUD____________C tST LANTA, GA. ACS
				

